# Differences in Upper-Body Peak Force and Rate of Force Development in Male Intermediate, Advanced, and Elite Sport Climbers

**DOI:** 10.3389/fspor.2022.888061

**Published:** 2022-06-28

**Authors:** Vegard Vereide, Vidar Andersen, Espen Hermans, Jarle Kalland, Atle Hole Saeterbakken, Nicolay Stien

**Affiliations:** ^1^Faculty of Education, Arts and Sports, Western Norway University of Applied Sciences, Sogndal, Norway; ^2^Faculty of Education, Arts and Sports, Western Norway University of Applied Sciences, Bergen, Norway

**Keywords:** climbing, finger strength, performance, testing, rate of force development (RFD)

## Abstract

The aim of this study was to investigate the difference in climbing-specific strength and rate of force development (RFD) between intermediate, advanced, and elite male sport climbers. Seventy-eight male climbers were recruited and divided into groups based on the International Rock Climbing Research Association (IRCRA) numerical (1–32) grading system (intermediate (10–17) group (IG; *n* = 28)), advanced (18–23) group (AG; *n* = 30) and elite (24–27) group (EG; *n* = 20). Peak force (*F*_peak_) and average force (*F*_avg_) were measured while performing an isometric pull-up on a 23 mm thick campus rung. RFD was calculated from the onset of force to maximal peak force. The elite group performed better in all test parameters than the advanced (*F*_peak_: 39.7%, ES = 1.40, *p* < 0.001; *F*_avg_: 45.6%, ES = 4.60, *p* < 0.001; RFD: 74.9%, ES = 1.42, *p* = 0.001) and intermediate group (*F*_peak_: 95.7%, ES = 2.54, *p* < 0.001, *F*_avg_: 131.1%, ES = 5.84, *p* < 0.001, RFD: 154.4%, ES = 2.21, *p* = 0.001). Moreover, the advanced group demonstrated greater *F*_peak_ (40.1%, ES = 1.24, *p* < 0.001), *F*_avg_ (59.1%, ES = 1.57, *p* < 0.001) and RFD (45.5%, ES = 1.42, *p* = 0.046), than the intermediate group. Finally, climbing performance displayed strong correlations with *F*_peak_ (*r* = 0.73, *p* < 0.001) and *F*_avg_ (*r* = 0.77, *p* < 0.001), and a moderate correlation with RFD (*r* = 0.64, *p* < 0.001). In conclusion, maximal force and RFD in a climbing specific test are greater among climbers on higher performance levels. Independent of climbing level there is a moderate-to-strong association between maximal and rapid force production and climbing performance.

## Introduction

Competitive climbing is divided into the three disciplines lead climbing (sport climbing), bouldering, and speed climbing, with sport climbing being the most practiced discipline (Saul et al., 2019). Generally, both in the climbing community and in research, the self-reported grade performed on a sport climbing route or boulder problem indicates climbing ability (Draper et al., 2011). A variety of climbing ability groups have been examined in climbing research on (Baláš et al., 2014; Hermans et al., 2017; Levernier and Laffaye, 2019, 2021). The International Rock-Climbing Research Association (IRCRA) recommend for research to use standardized climbing ability levels with the following classifications: lower grade, intermediate, advanced, elite, and higher elite (Draper et al., 2015). The performance ability levels are valuable to climbing research by allowing for standardization of the classification of climbers within a study, and to compare data between studies.

Several recent studies support finger strength and rate of force development (RFD) being significantly different between IRCRA ability groups and important predictors of climbing performance (Giles et al., 2020; Levernier and Laffaye, 2021; Rokowski et al., 2021; Stien et al., 2021b). For example, Giles et al. (2020) and Rokowski et al. (2021) showed that higher-elite and elite climbers had higher finger strength compared to elite and advanced climbers, respectively. Moreover, Torr et al. (2020) and Baláš et al. (2014) found significant correlations (*r* = 0.42–0.79) between relative finger strength and climbing performance. Finally, Stien et al. (2021b) reported that male elite climbers had significantly higher peak finger strength and RFD than advanced and intermediate climbers. Of note, the study is limited by a skewed distribution of climbers within performance levels. There is, to the authors' knowledge, no study that has used the average grade of the IRCRA ability groups when comparing finger strength and RFD between groups.

With the recent inclusion of climbing in the Olympic program, the demand for sound methods for testing athletes is increasing. The 2021 IRCRA study (Draper et al., 2021) included a suggestion of tests examining climbing performance. However, these tests do not necessarily represent valid measurements or reliable outcomes across climbing skill levels. Therefore, more knowledge about objective measurements that predict and differentiate between climbing performance levels is warranted. Although finger strength and RFD are considered important predictors of sport climbing performance (Laffaye et al., 2016; Michailov et al., 2018; Giles et al., 2020; Stien et al., 2021b), very few studies have compared these metrics across several levels of climbers. Therefore, the main aim of this study was to examine maximal isometric force (*F*_peak_), average force for 2 s (*F*_avg_), and RFD in male intermediate, advanced, and elite level sport climbers. It was hypothesized that *F*_peak_, *F*_avg_ and RFD would increase with increasing sub-class levels and that there would be a significant relationship between *F*_peak_, *F*_avg_ and RFD and climbing performance.

## Methods

### Experimental Approach to the Problem

A cross-sectional design was used to examine maximal isometric strength and RFD, and their association to climbing performance in climbers at three different performance levels. The testing included one visit to the laboratory for all participants.

### Subjects

Seventy-eight male sport climbers at different performance levels volunteered for this study. A criterion for the study was that the average performance level for the three groups should match the average IRCRA grade for intermediate (13.5), advanced (20.5) and elite (25.5) ability level. The participants had to be strong enough to perform a pull-up on the 23-millimeter (mm) thick rung, free of injuries, and have a minimum self-reported climbing ability of IRCRA grade 10 [French grade (f)5+] in the last 6 months. Based on the recommendations by Draper et al. (2015), the intermediate group (IG; =28) was defined as IRCRA 10–17 (f5+-f7a), the advanced group (AG; *n* = 30) as IRCRA 18–23 (f7a–f8a), and the elite group (EG; *n* = 20) as IRCRA 24–27 (f8a+-f8c). All participants were informed orally and in writing about the procedures and the potential risks and benefits of participating in the testing. A written consent had to be signed before data collection began. The study conformed to the latest revision of the Declaration of Helsinki and was conducted in accordance with the ethical guidelines of the Western Norway University of Applied Sciences. The preservation of the participants' safety and privacy was reviewed by the Norwegian Centre for Research Data.

### Testing Procedures

The participants had to refrain from high intensity climbing related or upper body training in the 48 h prior to testing. The testing started with a short questionnaire about age, climbing experience, prioritized discipline, maximal self-reported redpoint grade last 6 months, and if they had injuries that could affect performance in the testing. Anthropometrics were gathered using a Tanita bioelectric impedance scale (MC 780MA S, Tokyo, Japan) and a wall-mounted measuring tape.

To prepare for physical testing, a 15-min light-to-moderate warm-up was performed on a bouldering wall. The participants were instructed to start with easy bouldering (two-to-three number grades below their limit) and to progressively increase the intensity but to avoid fatigue. After 5 min of rest the participants were familiarized with the isometric test set-up and informed about how the procedures were performed. Participants were given three practice attempts with a sub-maximal effort before the experimental testing began.

**Figure 1:**
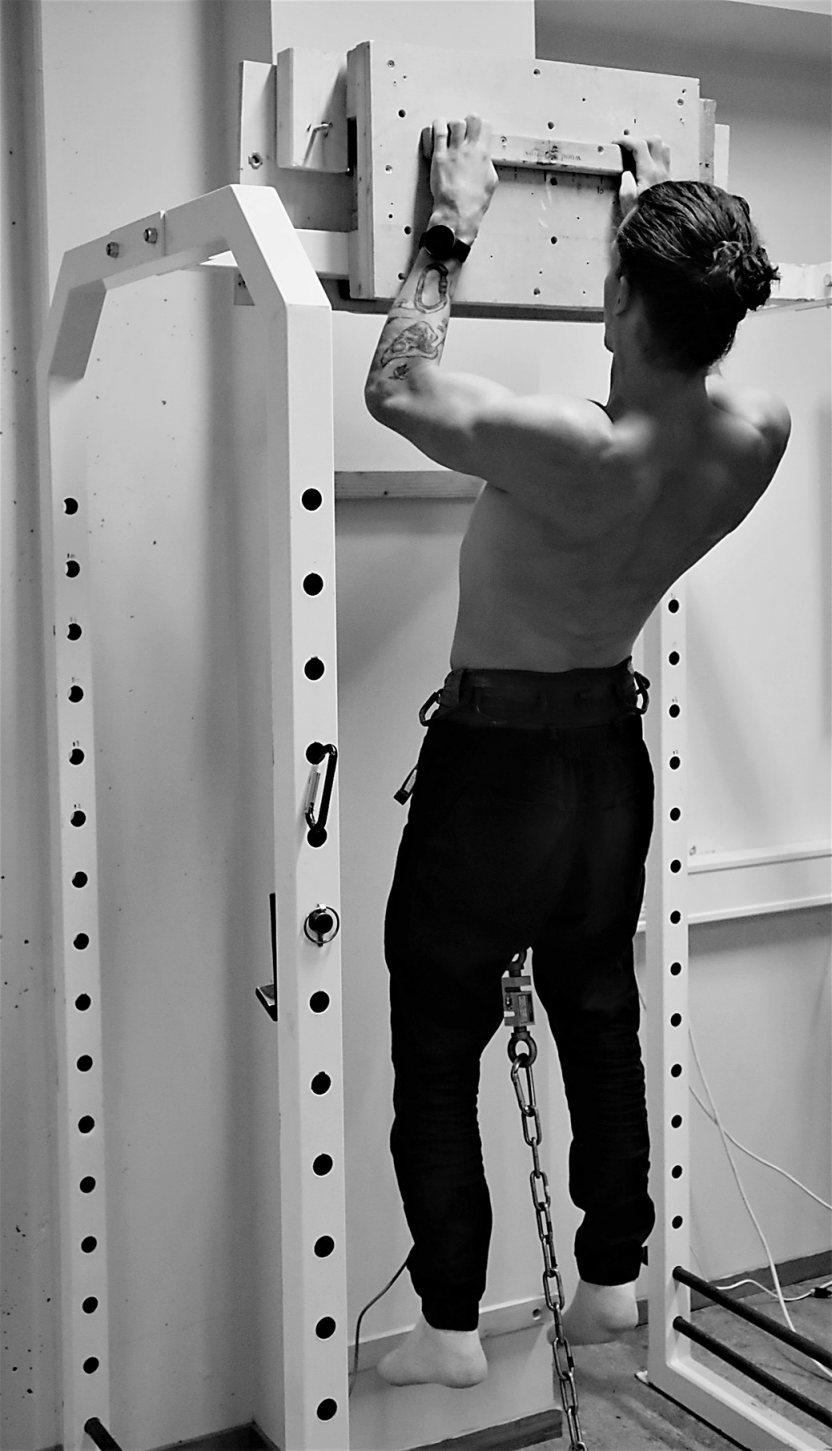
Image showing a participant performing the isometric pull-up test.

The maximal voluntary isometric contraction (MVIC) in the pull-up exercise was conducted on a 23 mm thick wooden rung with a fixed 90° elbow joint angle (measured with a goniometer) and a half-crimp grip with a passive thumb while anchored to a force cell at the floor with a static cord (Figure 1). The participants were allowed to use chalk on their fingers and hands during the testing. The rung was brushed between trails to avoid reduced friction due to excessive chalk left from previous tests. The cord had to be completely taut before the test began and no kipping with the legs or creating a countermovement were allowed. The force-time curves criteria have been described previously (Stien et al., 2021a). The participants had to hang still on the rung (no more than ±5 N fluctuation in force for 1,000 ms) before exerting maximal force (Stien et al., 2021b). The MVIC and RFD were measured using a force sensor sampling at 200 Hz (Ergotest Innovation A/S, Porsgrunn, Norway) and analyzed using commercial software (MuscleLab v.10.4, Ergotest Innovation A/S, Porsgrunn, Norway). The MVIC tests included three different parameters: (1) peak isometric force (*F*_peak_), (2) average isometric force across 2 s (*F*_avg_), and (3) RFD. The RFD was calculated from onset to peak force (Stien et al., 2021b). Three attempts separated by 3 min of rest were given and the attempt with the highest values was used in the analyses. Absolute values were used since (1) the body mass appears to be accounted for when the test is performed hanging, and (2) near identical results were found using absolute and relative values.

### Statistical Analyses

All statistical analyses were performed in SPSS (IBM Corp. Released 2020. IBM SPSS Statistics for Windows, Version 27.0. Armonk, NY: IBM Corp) and statistical significance was accepted at *p* < 0.05. A Shapiro-Wilk test revealed that IRCRA level (*p* = 0.031), years of experience (*p* < 0.001), and RFD (*p* = 0.002) were not normally distributed, whereas the remaining variables were (*p* = 0.059–0.739). To compare the *F*_avg_ and *F*_peak_ between groups, a one-way ANOVA with Bonferroni *post-hoc* correction was used. The RFD was analyzed using a Kruskall-Wallis test with Bonferroni *post-hoc* tests. The Cohen's *d* effect size (ES) for the differences between the climbing levels was calculated as the means divided by the pooled standard deviation. An ES <0.2 was considered trivial, between 0.2 and 0.5 as small, between 0.5 and 0.8 as moderate and above 0.8 as large (Cohen, 1998). The correlation between climbing performance and the three performance variables *F*_avg_, *F*_peak_, and RFD was assessed using Spearman's rho. Correlation values <0.3, between 0.3 and 0.5, between 0.5 and 0.7, and >0.7 were considered very weak, weak, moderate, and strong, respectively (Cohen, 1998).

## Results

### Anthropometrics

Age, height, and body mass were not different between the groups (*F* = 0.344–1.321, *p* = 0.273–0.710). Relative fat mas (% of body mass) was significantly different between groups (*F* = 5.349, *p* = 0.007) and *post-hoc* tests showed that the intermediate group had a greater fat mass than the advanced group (ES = 0.77, *p* = 0.007). No differences in fat percentage between the intermediate and elite groups (ES = 0.75, *p* = 0.097) or between the elite and advanced groups were observed (ES = 0.18, *p* = 1.000; see Table 1).

**Table 1:** Anthropometric data, climbing experience, weekly climbing sessions and self-reported climbing ability (IRCRA scale).

	**Intermediate (*n* = 28)**	**Advanced (*n* = 30)**	**Elite (*n* = 20)**
Age (year)	26.7 ± 6.2	29.0 ± 6.9	28.2 ± 7.2
Height (cm)	178.8 ± 7.3	180.1 ± 6.9	180.3 ± 6.3
Body mass (kg)	74.6 ± 9.3	72.5 ± 7.5	70.9 ± 6.2
Fat mass (%)	14.3 ± 3.5^*^	11.4 ± 4.0	12.0 ± 2.6
Year of climbing experience	5.0 ± 4.8	8.0 ± 5.8	13.7 ± 6.4^*^^†^
Weekly climbing sessions	2.4 ± 1.2	3.2 ± 1.0	4.4 ± 1.0^*^^†^
Red-point (IRCRA grade)	14.0 ± 1.7	20.5 ± 1.3^†^	25.4 ± 1.1^*^^†^

* *Greater than advanced (p < 0.01)*.

† *Greater than intermediate (p < 0.01)*.

### Climbing Experience, -Volume, and -Performance

Climbing experience (years) was different between groups (*F* = 14.147, *p* < 0.001). *Post hoc* tests revealed no difference between the intermediate and advanced groups (ES = 0.57, *p* = 0.140). The elite group had significantly longer experience than the intermediate (ES = 1.55, *p* < 0.001) and advanced groups (ES = 0.94, *p* = 0.002). The number of weekly climbing sessions was significantly different between groups (*F* = 14.036, *p* < 0.001). No difference was found between the intermediate and advanced groups (ES = 0.73, *p* = 0.140). The elite group had a significantly higher number of weekly sessions than intermediate (ES = 1.82, *p* < 0.001) and advanced groups (ES = 1.20, *p* = 0.002). Self-reported climbing ability (IRCRA) was significantly different between all groups (*F* = 14.147, *p* < 0.001; see Table 1).

### Force and RFD

For the elite group, all three variables were significantly greater than the advanced and intermediate groups (*p* < 0.001 for both). The elite group demonstrated greater *F*_peak_ (39.7%, ES = 1.40, *p* < 0.001), *F*_avg_ (45.6%, ES = 4.60, *p* < 0.001), and RFD (74.9%, ES = 1.30, *p* < 0.001) than the advanced group and the intermediate group (*F*_peak_: 95.7%, ES = 2.54, *p* < 0.001; *F*_avg_: 131.1%, ES = 5.84, *p* < 0.001; RFD: 154.4%, ES = 2.21, *p* < 0.001). The advanced group demonstrated greater *F*_peak_ (40.1%, ES = 1.24, *p* < 0.001) and *F*_avg_ (59.1%, ES = 1.57, *p* < 0.001) than the intermediate group, whereas RFD was not significantly different between the two groups (45.5%, ES = 1.42, *p* = 0.057; Table 2).

**Table 2:** Absolute values from isometric pull-ups, percent difference between groups.

	**Intermediate (*n* = 28)**	**Advanced (*n* = 30)**	**Elite (*n* = 20)**
*F*_peak_ (N)	353 ± 105	494 ± 122^*^	690 ± 155^†^
*F*_avg_ (N)	227 ± 88	361 ± 82^*^	524 ± 126^†^
RFD (N·s^−1^)	948 ± 357	1379 ± 721	2412 ± 865^†^

* *Higher than the intermediate group (p < 0.01)*.

† *Higher than the intermediate and advanced groups (p < 0.01)*.

### Correlations

**Figure 2:**
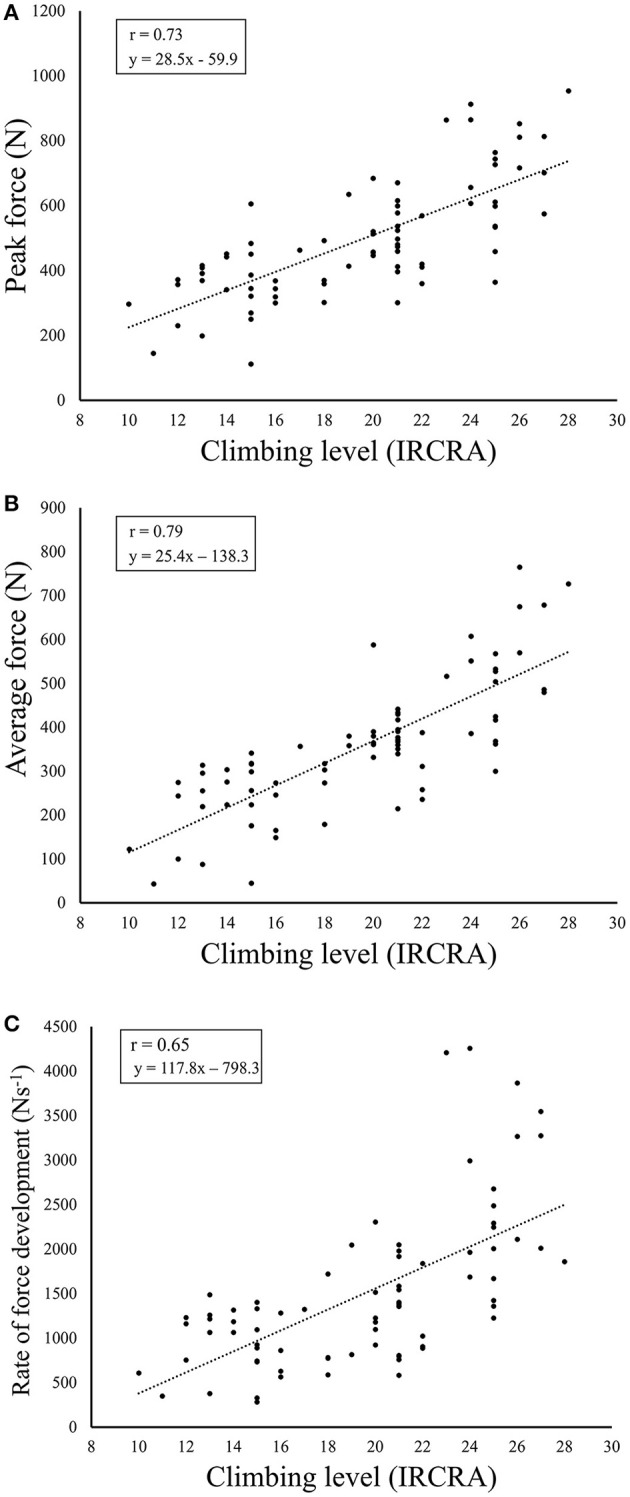
Scatter plots with imbedded Spearman's rho (*r*) values for the correlation between the International rock climbing research association (IRCRA) climbing performance level and **(A)** peak- and, **(B)** average force output in Newtons (N), and **(C)** rate of force development.

A strong correlation with climbing performance was found for *F*_peak_ (*r* = 0.73, *p* < 0.001) and *F*_avg_ (*r* = 0.79, *p* < 0.001), while a moderate correlation was found between RFD and climbing performance (*r* = 0.65, *p* < 0.001; Figures 2A–C).

## Discussion

This study compared the peak and average force outputs, as well as the RFD during an isometric pull-up performed on a 23 mm thick rung in intermediate, advanced, and elite sport rock climbers. In accordance with the hypothesis, *F*_avg_, *F*_peak_ and RFD were different between the three groups with the higher performance levels displaying greater maximal and rapid force production.

The *F*_peak_ increased similarly between the three groups, with 40.1% (ES = 1.24) from intermediate to advanced and 39.7% (ES = 1.40) from advanced to elite. These results contrast with the findings by Stien et al. (2021b) who found no difference in *F*_peak_ between the intermediate and advanced groups and speculated that maximal strength was less important than other factors (e.g., climbing technique) when transitioning between the two levels. However, the findings by Stien et al. (2021b) are challenged by the fact that the intermediate group had an average red-point grade of IRCRA 15.8 which is close to the advanced classification of ≥18. The current study might provide a clearer picture of the differences between the groups as the average red-point grades within the groups was close to the averages of each performance level according to the IRCRA classifications (Draper et al., 2015).

In contrast with the *F*_peak_, the percentage difference in *F*_avg_ between the advanced and elite groups (45.6%) was smaller than the difference between the advanced and intermediate climbers (59.1%). This could be explained by the difference in hold types (smaller sizes and less positive shapes) that often characterize routes graded IRCRA ≥18 (advanced) compared to the intermediate grades. Importantly, the observed effect sizes suggest that the difference between advanced and elite climbers (ES = 4.60) was more meaningful than that between intermediate and advanced (ES = 1.57). This trend is supported by the findings for RFD which displayed a 75% difference (ES = 1.30, *p* < 0.001) between advanced and elite groups, as well as a non-significant (p = 0.057) tendency for a 45% difference (ES = 1.42) between the intermediate and advanced groups. This could indicate that RFD becomes an increasingly important limiting factor for climbing performance when the elite grades are reached (IRCRA ≥24 for men). One potential explanation for this could be that the demands of the elite grades (e.g., steep routes and long distances between holds) challenge the RFD more directly than the *F*_avg_ through high-intensity movements similar to those observed in bouldering (White and Olsen, 2010). Previous findings highlighting the importance of RFD for climbing- (Levernier and Laffaye, 2021; Stien et al., 2021b) and bouldering-performance (Fanchini et al., 2013; Stien et al., 2019) support this speculation.

The validity of the relationship between *F*_peak_, *F*_avg_ and climbing performance is further supported by the strong correlation revealed for these parameters in this and previous studies (Baláš et al., 2014; Torr et al., 2020). Interestingly, the correlation between absolute strength and climbing performance in this study (*r* = 0.73–0.79) was similar to that observed using relative strength (*r* = 0.79) by Baláš et al. (2014). The current test set-up likely accounts for body mass to a greater degree since the test is performed hanging rather than standing. The association between climbing performance and RFD was lower (*r* = 0.64), which could imply that maximal strength is more important than RFD strength for climbing performance when analyzed irrespective of climbing performance level. This novel finding should be considered when examining climbers as the relatively wide ranges within groups (e.g., IRCRA 18–23 for the advanced classification) could hide potential differences between levels when the exact IRCRA grade is neglected.

For anthropometric variables, the only between-groups difference was found for relative fat mass between the intermediate and advanced groups, with the intermediate climbers having higher fat mass than the advanced climbers. Since no further differences were observed, it cannot be concluded that this metric has a meaningful impact on performance among climbers. This speculation is supported by previous research concluding that fat mass has a low predictive power for climbing performance (Laffaye et al., 2016). More interestingly, years of experience and number of weekly sessions were notably greater among the elite climbers than the other two groups, whereas no differences were found between the intermediate and advanced groups. Combined, these findings suggest that the magnitudes of training and climbing experience may be crucial factors for improving climbing performance. This speculation is supported by Mermier et al. (2000) who concluded that trainable factors were predictive of climbing performance, whereas specific anthropometric characteristics are less important to excel in climbing performance.

The reader should consider some potential limitations of this study when interpreting the findings. First, only male climbers were included, and the results may not be generalizable to females. Likewise, it is not certain that the findings would be similar if other grip positions or hold types were tested. No separate familiarization session was performed. Instead, several practice attempts were given, as well as three attempts in the experimental test to ensure that the optimal performance was measured. Still, we cannot exclude the possibility that a familiarization session could have improved the test performance. Moreover, since maximal and rapid force production were measured in the same attempts, it is possible that neither was optimized. Indeed, current recommendations (Maffiuletti et al., 2016) suggest performing separate attempts focusing on either reaching peak force as fast as possible (i.e., RFD focus) or reaching the highest possible force (i.e., maximal strength focus). We chose to focus on both parameters in all attempts to avoid fatigue by reducing the number of attempts that had to be conducted. Finally, it should be noted that climbing performance was not assessed directly. Importantly, self-reported climbing grades have previously been demonstrated as reliable and useable in scientific research (Draper et al., 2011).

In conclusion, maximal strength and RFD measured in an isometric pull-up on a 23 mm thick rung was able to differentiate between climbers performing on an intermediate-to-elite level and there were moderate to strong correlations between maximal strength and RFD and climbing performance. The results suggest that increases in maximal strength and RFD of the fingers are required to advance in performance, both within and between different climbing levels. To the authors' best knowledge, this is the first study to compare the strength across the three groups with averages adjusted to correspond to the IRCRA level average.

## Data Availability Statement

The raw data supporting the conclusions of this article will be made available by the authors, without undue reservation.

## Ethics Statement

The studies involving human participants were reviewed and approved by Norwegian Centre for Research Data. The patients/participants provided their written informed consent to participate in this study.

## Author Contributions

VV and NS wrote the original draft. All authors contributed to the conceptualization, data collection, and critical revision of the first draft. All authors contributed to the article and approved the submitted version.

## Conflict of Interest

The authors declare that the research was conducted in the absence of any commercial or financial relationships that could be construed as a potential conflict of interest.

## Publisher's Note

All claims expressed in this article are solely those of the authors and do not necessarily represent those of their affiliated organizations, or those of the publisher, the editors and the reviewers. Any product that may be evaluated in this article, or claim that may be made by its manufacturer, is not guaranteed or endorsed by the publisher.
